# Meningeal carcinomatosis diagnosed during stroke evaluation in the emergency department

**DOI:** 10.1186/1865-1380-4-52

**Published:** 2011-08-09

**Authors:** Derek R Cooney, Norma L Cooney

**Affiliations:** 1 Department of Emergency Medicine and Undersea and Hyperbaric Medicine, SUNY Upstate Medical University, EMSTAT Center/550 East Genesee, Syracuse, New York 13202, USA; 2 Department of Emergency Medicine and Undersea and Hyperbaric Medicine, SUNY Upstate Medical University, 750 East Adams Street, Syracuse, NY 13210, USA

## Abstract

A 70-year-old female presented to the emergency department with a 3-day history of intermittent dysphasia and right facial droop. Computed tomography (CT) and magnetic resonance imaging (MRI) were obtained, and the patient was found to have meningeal carcinomatosis, also known as leptomeningeal metastases. Meningeal carcinomatosis is a rare metastatic complication of some solid tumors and hematopoietic neoplasms, and has a median survival rate of 2.4 months. The role of the emergency physician is to appropriately diagnose this condition, treat emergent side effects, provide symptomatic relief, and ensure multi-disciplinary management.

## Background

Meningeal carcinomatosis (MC), also known as leptomeningeal metastases, is a rare metastatic complication of some solid tumors and hematopoietic neoplasms [1]. Incidence in patients with a primary solid tumor is 4-15% [2]. The median survival rate is around 2.4 months, with a rate of 2.3 months for solid tumors and 4.7 months for hematopoietic tumors [3]. The most commonly associated primary solid tumors are breast carcinoma (12-34%), lung carcinoma (10-26%), and melanoma (17-25%) [2]. Although most patients found to have MC have a previously diagnosed primary neoplasm, in a study by Clarke and colleagues published in 2010 as many as 9-16% of patients were thought to be disease free until diagnosed with MC [3].

## Case presentation

A 70-year-old female presented to the emergency department with a 3-day history of intermittent dysphasia and right facial droop. The patient had just returned from an overseas flight the day prior to the onset of symptoms. There was no history of headache, nausea/vomiting, or dizziness. Upon arrival, the patient had a generalized tonic-clonic seizure that responded to benzodiazepines. On examination, vital signs were blood pressure 138/76, P 124, R 23, and O_2 _Sat 100% RA. The patient was post-ictal, but became arousable and alert during the initial evaluation. Cardiac exam showed an irregularly irregular rhythm. She had an expressive aphasia with significant weakness to the right upper and lower extremity and right facial droop. The patient was unable to name objects or answer yes and no questions. Laboratory tests were unremarkable. A non-contrast CT head revealed lytic lesions of the skull and an abnormality of the brain. MRI of the brain with contrast showed vasogenic edema in the left frontoparietal region, dural thickening, and lytic/blastic lesions in the skull.

The approach to the patient with altered mental status includes a broad differential diagnosis including infectious, neurologic, and toxicologic causes. In this case, the presentation and history direct the physician to a neurologic etiology. The CT of the head confirmed the presence of a structural brain abnormality. MRI of the brain confirmed the diagnosis of meningeal carcinomatosis, and the edema finding provided some evidence of a brain tumor as the primary neoplasm.

In light of the MRI findings and her seizure activity, she was given an anti-epileptic drug to prevent further seizures and dexamethasone. Dexamethasone has been shown to decrease intracranial pressure and cerebral edema in cases of brain tumors [4]. Patients may respond to intrathecal chemotherapy and external beam radiation in some cases. Systemic chemotherapy may be an option in some cases. However, stabilization and symptomatic care are the immediate goals in the ED, and the patient improved with anti-epileptic drugs (AEDs), steroids, and narcotics prior to admission.

## Discussion

This case of meningeal carcinomatosis is somewhat unique in its presentation. The patient was not known to have cancer at the time of her presentation, and her symptoms were focal and stroke-like. It is uncommon for MC to be diagnosed in patients without a previous diagnosis of cancer [3]. In the review by Taillibert et al. facial weakness was an associated initial finding in only 25% of patients with MC, and seizure was only noted in 14% [2]. The fact that the patient exhibited aphasia, extremity weakness, and facial droop is likely secondary to the patient's brain mass and edema.

Although MC is usually a secondary metastatic disease from solid tumors, like cancer of the breast or lung, direct spread from primary CNS tumors is possible [2]. Focal findings with asymmetry are a poor prognostic sign, and MRI of the brain should be followed up with imaging of the entire neuroaxis. Lumbar puncture may also be considered and will likely yield abnormal opening pressure, cell counts, glucose, protein, or cytology. Cytology can be negative in up to 40-50% on initial lumbar puncture [2]. Lumbar puncture should be performed only after MRI if possible to avoid false-positive enhancement at the site.

Computed tomography is 1.5-2 times less specific and sensitive than MRI. Contrast-enhanced MRI is preferred, and larger doses of gadolinium are thought to reduce the false-negative rate [2]. Hydrocephalus and contrast enhancement of the meninges and sulci are common findings. Other testing, such as CSF flow studies and PET scans are not appropriate for the ED setting. Meningeal biopsy and non-specific biomarkers are sometimes obtained during the inpatient evaluation.

Emergency department management of seizure includes benzodiazepines and AEDs. However, AEDs are not thought to be needed on a prophylactic basis. Treatment of MC-associated headache, neck, and back pain should include analgesics, but may also include steroids. Alternatives to standard analgesics may be appropriate in patients who can be managed as an outpatient. Alternative pain management drugs like amitriptyline, gabapentin, carbamazepine, or benzodiazepines may be prescribed for chronic pain. Acute worsening of headache, neck pain, or back pain could be related to worsening complications, such as obstructive hydrocephalus, edema, or impingement of nerve roots or the spinal cord. Careful exam and history should be used to guide the clinician in determining the need for additional imaging or other diagnostics.

Despite intra-reservoir or intravenous chemotherapy, survival is merely 20-23 weeks [5]. External beam radiation may be used to control tumor growth at areas of impingement or severe pain. Chemotherapy and radiotherapy for MC are considered palliative in most cases.

## Conclusion

Meningeal carcinomatosis is a malignancy with poor survival rates. The primary sites of this type of metastatic cancer are typically breast and lung, but may include other solid tumors as well as hematopoietic tumors. Most patients diagnosed with MC will already be known to have cancer, but around 9-16% will not, until the time that MC is diagnosed. Despite aggressive chemotherapy and radiotherapy, survival is limited. The role of the emergency physician is to appropriately diagnose the condition and arrange for multi-disciplinary management after stabilization and pain management.

## Consent

Consent was obtained for publication of the details of this case and for publication of associated radiographic images.

## Competing interests

The authors declare that they have no competing interests.

## Authors' contributions

NC participated in the care of the patient and provided case details. DC prepared images, reviewed reports, and performed literature searches. Both DC and NC reviewed the literature and provided authorship of the text of this manuscript.

**Figure 1:**
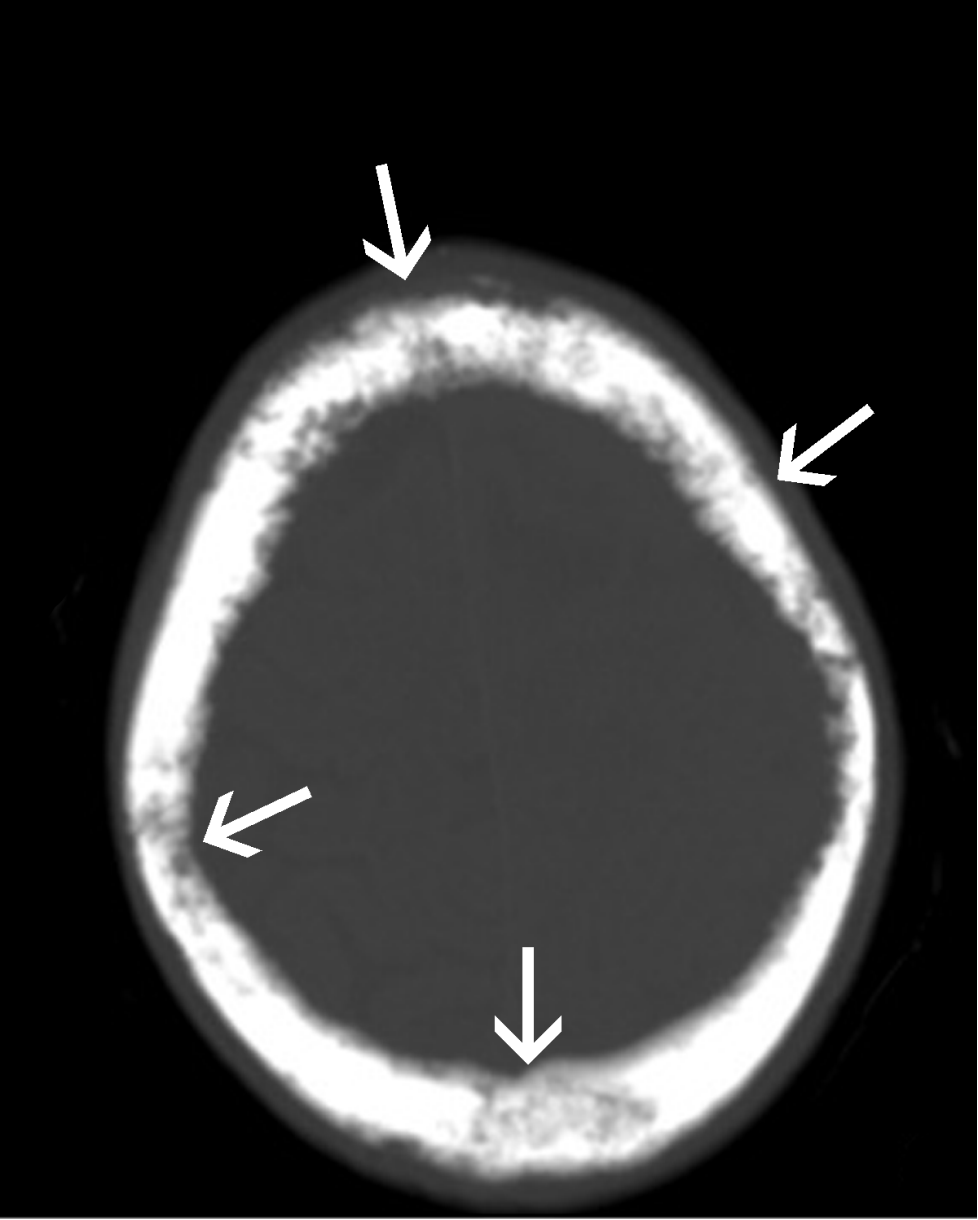
**CT scan revealing diffuse lytic/blastic lesions of the skull - arrows**.

**Figure 2:**
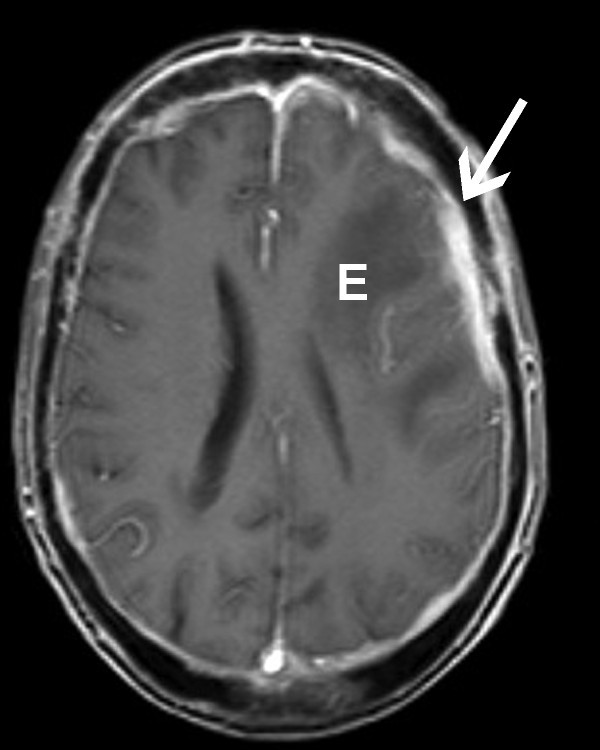
**MRI with contrast**. Vasogenic edema - E. Nodular dural thickening - arrow.
